# Musical Expertise and the Ability to Imagine Loudness

**DOI:** 10.1371/journal.pone.0056052

**Published:** 2013-02-27

**Authors:** Laura Bishop, Freya Bailes, Roger T. Dean

**Affiliations:** 1 MARCS Institute, University of Western Sydney, Penrith, New South Wales, Australia; 2 Music Department, University of Hull, Hull, United Kingdom; UNLV, United States of America

## Abstract

Most perceived parameters of sound (e.g. pitch, duration, timbre) can also be imagined in the absence of sound. These parameters are imagined more veridically by expert musicians than non-experts. Evidence for whether loudness is imagined, however, is conflicting. In music, the question of whether loudness is imagined is particularly relevant due to its role as a principal parameter of performance expression. This study addressed the hypothesis that the veridicality of imagined loudness improves with increasing musical expertise. Experts, novices and non-musicians imagined short passages of well-known classical music under two counterbalanced conditions: 1) while adjusting a slider to indicate imagined loudness of the music and 2) while tapping out the rhythm to indicate imagined timing. Subtests assessed music listening abilities and working memory span to determine whether these factors, also hypothesised to improve with increasing musical expertise, could account for imagery task performance. Similarity between each participant’s imagined and listening loudness profiles and reference recording intensity profiles was assessed using time series analysis and dynamic time warping. The results suggest a widespread ability to imagine the loudness of familiar music. The veridicality of imagined loudness tended to be greatest for the expert musicians, supporting the predicted relationship between musical expertise and musical imagery ability.

## Introduction

Most of the sounds people encounter in their daily lives are dynamic. From music to speech to environmental noise, sounds change throughout their duration in terms of intensity, spectrum and frequency. Mental imagery may aid integration of this transient auditory information during perception as well as aiding in planning during sound production. The extent to which loudness, a perceptual correlate of intensity [1], [2], [3], [4], is represented in imagery is the focus of this study. Loudness is of particular relevance to the dynamic nature of sound because of the significance *loudness change* has in both environmental [5] and musical contexts [6]. Imagining previously encountered sounds involves reconstructing a mental representation of them from information stored in memory, and while it is unclear whether loudness is a part of the memory trace for a single auditory event [7], perhaps a change in loudness is part of the memory trace of a sequence of events. If change in loudness rather than the loudness of individual auditory events is stored in memory, then it is likely that change in loudness rather than the loudness of individual auditory events can be imagined.

The principal aims of this study were to investigate whether the loudness of familiar classical music can be imagined and to assess the relationship between the veridicality of imagined loudness and musical expertise. Music is a naturalistic context for studying imagined relative loudness, as loudness plays a central role in musical expression [8]. Change in loudness emphasises structural boundaries [9] and is among the most often used parameters in the communication of affect, one facet of musical expression [2], [8], [10], [11]. The veridicality of imagined loudness change may depend on how precisely that loudness was perceived in the first place, whether a schema exists in memory that can facilitate retention, and how much detail can be accessed at the time the music is imagined. Expert musicians seem to have extraordinary memories for music [12]. If they encode and retrieve loudness information more effectively than do non-musicians [13], they may imagine it more veridically as well.

### Musical Imagery

Musical imagery is defined for the purposes of this research as the conscious experience of music in the absence of corresponding environmental input. Though pitch, duration [14], [15], [16], [17], [18], [19], [20] and timbre [21], [22] seem to be represented in musical images, the research on whether loudness is also represented in musical images is inconclusive [21], [23], [24]. Pitt and Crowder [21], for instance, presented people with sounded tones, prompted them to imagine those tones at either a loud or soft volume, then presented them with a second tone at either a loud or soft volume and asked them to compare the pitch of the two tones. Consistency in loudness between imagined and sounded tones had no priming effect on the participants’ judgements of pitch, and it was suggested that loudness may not be a component of auditory imagery. An alternative explanation is that changes in loudness can be accessed via imagery tasks, while the loudness of individual sound events cannot. If changes in loudness are imagined, this is less likely to be accessed in an imagery task in which tones are presented individually, separated by a period of silence, than in a task in which they are part of a musical sequence, or melody. In the current study, people were asked to imagine familiar passages of classical music, and the veridicality of imagined loudness change across sequences of notes was investigated.

Similarly inconclusive findings were reported by Intons-Peterson [24], who investigated imagery for the loudness of individual environmental sounds. It was predicted that the time needed to generate an auditory image of a sound would relate to the loudness of that sound. Though the time needed to compare two imagined sounds increased when the sounds were rated as being more dissimilar in terms of loudness, the time needed to generate a single auditory image was not significantly related to rated loudness of the to-be-imagined sound. It was concluded that loudness is an optional component of auditory imagery, available only under certain contextual demands. These conclusions were based on the assumption that people scan upwards from a loudness of ‘zero’ when imagining sounds. If this is not the case, then the length of time it takes to imagine sounds could be unrelated to their associated loudness, and such a task may not be the optimal measure for assessing imagined loudness.

A criticism of many imagery studies, such as that conducted by Intons-Peterson [24], is that they fail to provide sufficient evidence for participants having *imagined* a stimulus instead of merely reporting abstract knowledge about it [25]. This shortcoming has been pointed out by proponents of ‘descriptive’ theories of mental imagery, which, in contrast to ‘depictive’ theories, propose that images comprise a symbolic, language-like code instead of mirroring the form of their physical analogue [26], [27]. It is acknowledged that the mental representation of an object or event may comprise a combination of depictive and descriptive aspects at any one time, and that people may rely primarily on depictive imagery under some circumstances and descriptive knowledge under other circumstances. An aim of the present study was to test whether loudness change in music can be imagined depictively. A continuous response task was used that would have been extremely difficult to complete without the aid of depictive imagery. Participants were required to recall excerpts from well-known pieces of classical music under silent conditions, and to make continuous loudness judgements that were similar in direction and magnitude to the changes in acoustic intensity present in reference recordings. Attempting to complete the task by carrying out sequences of automatised or verbally-encoded action commands would have placed unreasonable demands on memory. A similar approach to designing an imagery task that would be nearly impossible to complete successfully without the aid of imagery is reported by Lucas, Schubert, and Halpern [28], who used a continuous response task to compare musicians’ emotional responses to imagined and sounded music.

Loudness change in sounded music has been shown to be a universally informative parameter of musical expression for listeners, regardless of their training and familiarity with the style of music being played [2], [29], [30]. Loudness change has been found to contribute reliably to listeners’ perceptions of emotional arousal, for instance [2], [30]. An aim of the current study was therefore to investigate whether the ability to imagine loudness is common to both trained musicians and non-musicians. While non-musicians can demonstrate accurate imagery for pitch and duration when reproducing the starting pitch or tempo of a familiar song [31], [32], there is also evidence that the ability to imagine these parameters improves with increasing expertise. Increased expertise is associated with an enhanced ability to hold a specific pitch in mind through a period of silence [18] and mentally compare pitches corresponding to lyrics in familiar songs [17], [19]. It is also associated with an enhanced ability to maintain accurate pitch and tempo in mentally continuing musical sequences following a short, sounded introduction [16]. If expert musicians imagine pitch and time more accurately than do novices, they may imagine other parameters, including loudness, more accurately too.

### Musical Expertise and Memory

Expertise is characterised by a maximisation of efficiency in the processing networks underlying performance on a specific set of tasks [33]. In the Western classical music tradition, performance expertise requires both technical and expressive mastery [12], [34]. Technical demands involve coordinating sequences of movements within a narrow margin of error, often at a rapid pace [34]. Technical proficiency lays the groundwork for musical expression, the systematic deviation from and addition of such features as loudness to the fixed pitch and time structure that distinguishes one piece of music from another, reflecting a specific interpretation of that music. Expression is one component of what can give music its aesthetic [35], emotional [36] and communicative qualities [36], [37]. Expert musicians are distinguished from non-experts by their ability to replicate their own expressive performances at will with near-perfect precision or, with little or no practice, alter their interpretation to produce an entirely different expressive profile [9].

Imagining familiar music involves a process of reconstruction. Prior knowledge of musical structure within the relevant musical tradition can support the veridical retention of some details and fill in where other details have been lost. Experts demonstrate superior memory for domain-relevant stimuli relative to novices, perhaps because they organise material in memory more effectively [12], [38]. Williamon and Valentine [13] observed differences in how highly-skilled musicians and novices structure memory while preparing a piece of music for performance. All musicians segmented the music during practice and performance, regularly stopping and starting at particular locations; these segments were understood to correspond to retrieval structures that participants were storing in memory. While novices often stopped and started at bars they found difficult, retrieval structures used by highly-skilled musicians tended to be hierarchical and corresponded more to formal music structure (e.g. thematic or phrase boundaries). Expressive loudness changes tend to relate predictably to specific structural features in the Western classical music tradition [6], [9], [36], such as phrase boundaries, and may be more likely to be imagined if reconstructed from a memory trace that preserves this underlying structural detail than if reconstructed from a memory trace that does not.

Though the differences in start and stop patterns observed in Williamon and Valentine’s [13] study suggest that expert and novice musicians may differ in how they organise memory for performed music, it is also possible that novices were hampered by a preoccupation with meeting technical demands rather than an inability to understand or remember structural detail or perform expressively. There is evidence to suggest that even non-musicians understand musical structure to a high degree, despite lacking the vocabulary needed to put their knowledge into words and the technical skills necessary to demonstrate it on music performance tasks. When imagining familiar music, for instance, non-musicians imagine sections, or chunks, that correspond to the underlying musical structure [39]. Non-musicians likewise differ little from skilled musicians in their ability to use implicit knowledge of structure when listening to music [40]. Furthermore, it remains unclear whether experts and novices differ in how they organise perceived music in memory and whether experts’ superior musical memories extend to an enhanced ability to reconstruct changes in loudness when imagining familiar music. In the present study, it was hypothesised that expressive loudness change can be imagined, and that the veridicality of this imagery improves with increasing musical expertise.

### Skills Associated with Musical Imagery Ability

In the design of any task used to assess the relationship between musical imagery ability and expertise, the skills musicians have refined explicitly through years of practice and training need to be taken into account. Abilities such as working memory span and music listening ability, or how closely a person can attend to sounded music, likely contribute to success on musical imagery tasks and should be taken into consideration.

The veridicality of imagery for familiar music depends, in part, on the strength of the memory trace from which it was reconstructed and, consequently, how effectively the music was encoded at the time it was sounded. This points to a potential correlation between musical imagery and listening abilities: a person who attends more closely to music while listening and encodes more detail may have a more veridical musical image than a person who attends less closely and encodes less detail. The relationships between attention paid while listening, detail encoded, and veridicality of imagery are not guaranteed, as it is possible to imagine detail that was not encoded and to perceive and attend to detail in sounded music without encoding it. However, research suggests that perceptual abilities improve with increasing expertise in music performance [21], [41], [42],[43],[44], and this improvement may be a contributing factor to experts’ superior musical imagery abilities. To avoid confounding the capacity to imagine music with the attention paid while listening and the detail encoded, listening ability was assessed in the present study to determine whether it could account entirely for performance on the imagery task.

The veridicality of imagined music may also depend on working memory capacity. The working memory system permits temporary storage and manipulation of information and is said to mediate mental imagery [45], [46]. Whether musical expertise is associated with improvements in such general cognitive abilities as working memory is a question of interest in the musical expertise literature [47], [48], [49]. The relationship between musical expertise and general working memory capacity is unclear, however. Theoretical accounts of expertise posit that more effective structuring of domain-relevant material in working memory, rather than greater general working memory capacity, enables experts to reliably outperform novices [38], [50]. While some researchers in the music domain have found expert and novice musicians to perform similarly on tasks assessing working memory capacity [48], [49], others have observed a greater verbal working memory capacity in trained musicians relative to non-musicians [47] In the present study, working memory span was also assessed to ensure that general memory abilities (i.e. not domain specific) could not account entirely for performance on the imagery task.

### Present Research

Musicians stress that it is important to be able to imagine the desired effects of their actions in order to produce them [51], implying that those who are better at performing music are likewise better at imagining it. Some research, also, suggests that musical imagery may partially compensate and enable performance or mental rehearsal in the absence of auditory or motor feedback [52], [53], [54]. Repp [23] found that skilled pianists only slightly attenuate their performance of expressive loudness, measured in terms of key velocity, when playing on a silent keyboard, compared to their performance under normal conditions. These pianists’ success at achieving some expressive loudness during silent performance suggests that loudness can be part of a mental image guiding performance and that, to a degree, this guiding image may compensate for the absence of auditory feedback. If expert musicians can imagine loudness, are they better able to do so than novice musicians and non-musicians? The present study investigated the abilities of expert musicians, novice musicians, and non-musicians to imagine loudness in well-known classical music. Participants were grouped according to their scores on the Ollen Musical Sophistication Index (OMSI) [55], [56], which categorises people as more or less musically sophisticated based on such factors as amount and level of formal training, composition experience, and practice and music listening habits. In most previous research on musical expertise, comparisons have been made between either expert and novice performers, or between musicians and non-musicians. Three skill groups, in contrast, were included in the present study to investigate the possibility that expertise groups differ asymmetrically in terms of imagery ability. Some musical skills, such as knowledge of how to read music notation, may develop earlier than other skills, such as the ability to communicate expression, in people learning to play an instrument. Imagery ability may be among those skills that develop early in the course of musical training, in which case novices would perform more like experts on imagery tasks than like non-musicians. Alternatively, imagery ability may be among those skills that develop later, in which case novices would perform more like non-musicians than experts.

Based on evidence that expert musicians imagine pitch and duration more accurately than novices or non-musicians [16], [18], [19] and organise musical information more effectively in memory [13], [57], [58], it was hypothesised that the veridicality with which loudness change can be imagined would increase as a function of musical expertise. Participants imagined short passages of well-known classical music while, in one condition, tapping out the rhythm, and in the other, adjusting a slider to indicate imagined loudness. Similarity between participant response profiles and original recording profiles was expected to increase as a function of expertise. Both loudness and tapping conditions were then repeated while participants listened to the same passages, so that listening ability could be assessed. Tapping data were collected and used as preliminary evidence that participants had recalled the correct passages of music. Working memory span was evaluated using an automated Operation Span Task (OSPAN) [59].

## Methods

### Ethics Statement

Written informed consent was obtained from all participants, and the study was approved by the University of Western Sydney Human Research Ethics Committee (Approval number H7740).

### Participants

Fifty-eight participants from a variety of musical backgrounds took part in the experiment. A subset were musically-untrained psychology students at the University of Western Sydney (UWS); the remainder had at least one year of formal music training and included students at UWS, the Sydney Conservatorium of Music, and University of Canberra, as well as professional musicians in the Greater Sydney area. Tertiles were calculated for the distribution of participant scores on the OMSI and these values were used to categorise the participants who met inclusion criteria for each stimulus (see Analysis) into three expertise groups. The least experienced, or “non-musician” groups (age *M = *21.0, *SD* = 5.0 across stimuli) reported an average of nine months of formal training (*SD* = 1.2). The moderately experienced, or “novice” groups (age *M* = 34.9, *SD* = 16.8) reported an average of 3.2 years of formal training (*SD* = 5.1). The most experienced, or “expert” groups (age *M* = 26.1, *SD* = 6.8) reported an average of 11 years of formal training (*SD* = 5.7). The novice group was older on average than either of the non-musician or expert musician groups. Age did not correlate significantly with any of the dependent measures, however, suggesting that age-related differences could not account for the results. Musically-trained participants had studied one or more of a range of instruments, including flute, guitar, piano, trumpet, viola, violin, and voice.

Psychology students at UWS received course credit for their participation; all others received a small travel reimbursement. All data for two additional participants were lost due to equipment failure. Loudness data for a third participant were also lost due to equipment failure, but timing data were retained and analysed.

### Stimuli

One excerpt was taken from each of three well-known pieces of Romantic-style orchestral music (*Blue Danube Waltz*, *Habanera*, and *Jupiter*). These pieces were selected from a larger pool based on a preliminary familiarity survey as well as length, the absence of lyrics, degree of dynamic variability, degree of rubato (expressive timing deviations), and the presence of an easily tapped melodic rhythm. On a scale of 1–5 (1 = “completely unfamiliar”; 5 = “very familiar”), mean preliminary familiarity ratings were (*N* = 6; age *M* = 27.6, *SD* = 5.5; years musical training *M* = 6, *SD* = 6.0): *Blue Danube Waltz* (5.0), *Habanera* (5.0), *Jupiter* (3.3). The passage from *Jupiter* was used as practice, and the passages from the *Blue Danube Waltz* and *Habanera* were used on experimental trials. The length and acoustic intensity range of the passages are reported in Table 1. Participants also completed the imagery and listening tasks for three additional passages (excerpts from *In the Hall of the Mountain King* (Grieg), *Sleeping Beauty Waltz* (Tchaikovsky), and *Swan Lake, Scène* (Tchaikovsky)), but due to the difficulty participants had recalling the rhythms of these passages, only participant response profiles for the *Blue Danube Waltz* and *Habanera* could be analysed for veridicality of imagined loudness, and only results for these two passages are presented here.

**Table 1:** Musical stimuli for imagery and listening tasks.

		Intensity (dB SPL)		
Selection	Extract	Range	Mean	SD
Carmen, Habanera (Bizet)	0′54–1′29	36.53	59.75	7.05
On the Beautiful Blue Danube (Strauss)	0′00–0′44	51.25	66.07	7.11
The Planets, Jupiter, The Bringer of Jollity (Holst)*	5′04–5′31	29.29	68.79	3.75

*Note.* Recording details: *Carmen, Habanera*, by St. Mark’s Philharmonic Orchestra. *On the Beautiful Blue Danube*, by the Orchester der Wiener Staatsoper and Anton Paulik (Brilliant Classics). *The Planets, Op. 32, Jupiter, The Bringer of Jollity*, by the Philadelphia Orchestra and Eugene Ormandy (RCA Victor).

* This stimulus was used for practice trials.

MP3 files for each stimulus were imported into *Audacity* and converted to.wav files in order to be readily compatible with MAX/MSP (sampling rate 44.1 kHz). The chosen passages were isolated and fades added where necessary to ensure that passages began and ended cleanly on phrase boundaries.

Intensity profiles of the reference recordings (dB SPL) were measured using the acoustic analysis software *Praat*. To establish note onset profiles, a time series of melody line interonset intervals (IOIs) was generated using *SonicVisualiser*. Note onsets were identified manually by two separate raters and Procrustes analyses of the similarity in contour between their resulting IOI profiles indicated high inter-rater reliability following standardization, *P*<0.0003 for both experimental passages. Procrustes analysis is used to calculate the degree of fit between two shapes with the effects of translation, scaling and rotation removed. It yields the statistic *P* which, when the analysis is applied to time series data, is a similar but more accurate representation of the fit between two data series than Pearson’s correlation. The closer *P* is to zero, the better the fit is between the two data series, with 1– *P* being comparable to *R* [60], [61].

### Equipment

Participants were seated in a quiet room at a MacBook (OS X 10.5.8), wearing Sennheiser HD 650 headphones. Imagery and listening tasks were run through a custom-designed patch in Max/MSP (5.1.9), which presented music stimuli and recorded participant response data. Tapping data were collected using a Roland Handsonic HPD 10 MIDI drumpad, and slider data were collected using an I-CubeX push v1.1. The slider (100 mm in length) was fixed to a plastic box that inclined away from the participant. Upwards movement, or movement away from the participant, indicated an increase in loudness, and downwards movement, or movement towards the participant, indicated a decrease in loudness. The top position represented the loudest point in the piece and the bottom position silence.

An automated version of the Operation Span Task (OSPAN) was presented to participants on a PC with Inquisit (see below).

### Design

A three-factor mixed model design was used, with expertise group acting as a between-subjects independent variable and task (imagery or listening) and condition (loudness or tapping) acting as within-subject independent variables.

### Procedure

The first phase of the experiment was designed to ensure that all participants were familiar with the same version of each well-known music stimulus and able to recall the passages. Participants were given a CD consisting of six short passages (3′32 total listening time) and labelled with the name of each piece, and instructed to listen to all six tracks at least twice a day, every day, for a week. They were told that this was a minimum and encouraged to listen to the CD as many times as they wanted during this period. Participants were told that the experiment was part of a study on familiarity and enjoyment of music. They were to rate liking and familiarity of each passage on 5-point scales each time they listened to it. The topic and aims of the experiment were withheld to prevent participants from selectively attending to specific parameters in the music or attempting to memorise it.

Participants came to the laboratory for the second phase of the experiment one day after completing their final listening assignment. They received general instructions, completed a musical background questionnaire (including all questions from the OMSI), and were asked to rate their familiarity with each stimulus. They then completed the imagery and listening tasks, followed by the OSPAN. The imagery tasks were always completed before the listening tasks. This ordering was to avoid influencing participants’ memory of the passages in the imagery task by sounding them immediately beforehand in the listening task, and to prevent them from memorising dynamics or timing patterns. Loudness and tapping trials were blocked separately within each task, with half the participants completing the loudness task first and half the tapping task. The order of passage presentation was randomised for each participant, irrespective of expertise group, within each of the four conditions. Participants completed the loudness and tapping tasks once for each passage under imagery conditions and once more for each passage under listening conditions, for a total of 24 trials (i.e. six excerpts including a practice excerpt; two tasks (loudness and tapping); two conditions (imagery and listening)).

At the start of each condition, participants received specific instructions about how to complete the task. As it was expected that non-musicians would have little or no experience in singling out individual parameters of music, such as loudness or timing, instructions for how to tap a rhythm and map out loudness were explained in detail. Participants were told to match tapping speed to changes in the speed of the music and, similarly, to match slider movements to speed, direction and degree of loudness change. Written and oral instructions were provided and were followed by a demonstration and practice trial with oral feedback from the experimenter to ensure that participants understood the task.

The following instructions were given for the imagined loudness task:

*When you indicate that you are ready to start, a few seconds of music will play, then rapidly fade to silence. As soon as you hear the music, adjust the slider to indicate the level of loudness you are hearing. When it fades out, continue adjusting the slider position to reflect the loudness of the music as you imagine its continuation, or “sing it in your head”.*

*Try to imagine the music at the speed you are used to hearing it. Don’t worry about forgetting what comes next or trying to get through as much as you can. Focus on hearing the music as clearly as possible in your head. Do not sing or hum the music aloud.*

During tapping condition practice trials, participants received visual feedback with the onset of each tap in the form of a blinking light on the computer screen so that they understood how much force was needed for the drumpad to detect their taps. This visual feedback was not given during the experimental trials. Participants were not instructed to relate the force of their tapping to the loudness of the sounded or imagined music, and tapping force was not recorded. The use of vocabulary that could be understood differently by musicians and non-musicians (e.g. expression, dynamics, tempo) was avoided.

#### Imagery task

In the loudness condition, brief instructions were presented on screen. Participants indicated that they were ready to begin the first trial by clicking the mouse on a start button. Two seconds later, a passage cue consisting of the first few seconds of the excerpt fading into silence was presented through their headphones (mean cue length 3.76 seconds; mean fade length 0.32 seconds). The volume was pre-set to a comfortable level, but participants were free to adjust it if they wished. The task was to map out changes in loudness of the imagined music by continuously adjusting the position of the slider. Slider position was recorded every 250 ms in MAX/MSP, since participants were unlikely to make meaningful loudness judgements at a finer resolution.

Participants began each trial with the slider in the bottom (silent) position, and were to adjust it as quickly as possible to match the loudness of the music they were hearing. When the cue faded out a few seconds later, they were to keep adjusting slider position to indicate the loudness changes in the imagined continuation of the passage. They were told to keep their hand on the slider at all times. A visual signal indicated the end of the trial; participants were told that this meant that they should be finished or almost finished imagining the passage. Along with the brief instructions that remained on the screen throughout the condition, this was the only visual information given. Participants were then allowed a short break and again indicated by clicking the mouse button when they were ready to begin the next trial.

A similar procedure was used in the tapping condition. Participants indicated when they were ready to begin, and two seconds later a cue was presented. The task was to tap out the rhythm of the main melody for the passage on the drumpad. The IOI between each tap was recorded in Max/MSP. Using the index or middle finger of their dominant hand, participants were to begin tapping as soon as possible after the cue began. When the music faded out, they were to keep tapping the melody while imagining the continuation of the passage. Again, a visual signal indicated the end of each trial, at which point participants were allowed a short break and indicated that they were ready to continue by clicking the mouse button. The experimenter remained in the room during testing to ensure that participants were not engaging in any unwanted production behaviour, such as vocalising the music. They were likewise cautioned against guessing, skipping sections or starting over mid-trial and, instead, were told to press a key to end a trial if they got lost or distracted while imagining the passage.

#### Listening task

As in the imagery task, brief instructions remained on the computer screen throughout each condition. Participants indicated when they were ready to begin by clicking the mouse on a start button, and two seconds later the music began. Instead of fading out after a few seconds, however, passages were played in their entirety. The task in the loudness condition was again to map out the loudness changes in the passage by continuously adjusting the slider. Participants began each trial with the slider in the bottom (silent) position and, when the music began playing, were to adjust it as quickly as possible to match loudness of the music they were hearing. They continued adjusting the slider until the passage concluded and a visual signal indicated the end of the trial, at which point they were allowed a short break before continuing.

The task in the tapping condition was again to tap out the rhythm of the main melody for each passage. Participants were to begin tapping the rhythm as soon as possible after the music began, and continue tapping throughout the duration of the trial. When the passage concluded, a visual signal indicated the end of the trial, just as in the imagery task, and participants were again allowed a short break before continuing.

#### Automated Operation Span Task (OSPAN)

An automated version of the Operation Span Task designed by Turner and Engle [59] was used to assess working memory. This task was selected from among the various available measures of working memory span on the basis of its high validity and reliability [62] and because it relies less than other measures on language abilities, which may also vary systematically as a function of musical expertise [63]. Participants received instructions and practice trials on the computer. During the task, equations containing two operations were presented in the centre of the computer screen (e.g. (24/3) +2). Participants indicated that they had mentally solved the equation by clicking the mouse button. A potential answer appeared immediately after their mouse click in the centre of the screen, and participants indicated whether this answer was correct by clicking the mouse on the appropriate response button (labelled ‘true’ and ‘false’). An upper-case letter was then displayed for one second in the centre of the screen. All numbers and letters were presented in a sans-serif font and the same font size (measuring.8 mm by.6 mm). Participants were required to verify equations as quickly as possible while maintaining a minimum accuracy of 85%. They were informed that after each set of between two and seven equations, they would be asked to recall the letters in the order they were presented. No access to pen and paper or other aids was permitted. A higher score on the OSPAN indicates greater working memory capacity.

### Analysis

It was hypothesised that participants in all expertise groups would be able to imagine the loudness of the passages, but that experts would imagine it more veridically than would either novices or non-musicians. To assess the veridicality of imagined loudness, each participant’s imagined loudness profile was compared with their listening loudness profile and the recording intensity profile (dB SPL). In other literature, listeners’ continuous response profiles have been compared with a measure of adjusted acoustic intensity thought to be more representative of the psychoacoustic parameter ‘loudness’ [64]; however, the benefits of using this measure in place of the unadjusted acoustic intensity measure used here have not been demonstrated [65]. Acoustic intensity was used as a reference in the current study because it has been shown to be the primary contributor to perceived loudness [3].

Table 2 lists the dependent variables and their definitions. One dependent variable indicated similarity between imagined loudness and recording intensity profiles (‘image-intensity similarity’ measure), one indicated similarity between imagined and listening loudness profiles (‘image-listening similarity’ measure), and one indicated ability to recall the experimental stimuli (‘recall’ measure) (Figure S1). Similarity between listening loudness and recording intensity profiles was assessed as well (‘listening-intensity similarity’ measure). These comparisons were made using time series analysis and dynamic time warping, as data points within profiles were not independent and correlations would have been uninformative. The three dependent variables and potential covariate measures of listening ability and working memory span then were examined to investigate the expected effects of expertise.

**Table 2:** Measures for evaluating imagined and listening loudness profiles.

Measure	Analysis definition	Function
Image-listening similarity	Difference between pre- and post-warping normaliseddistance separating shortened imagery andlistening profiles	Measure of how similarly participants rated sounded and imagined loudness in as much of each passage as they could remember
Recall	Difference between pre- and post-warping normalised distanceseparating full-length imagery and listening profiles	Measure of how much of each passage participants could imagine
Image-intensity similarity	Distance between the set of coefficients obtained by applyingthe parent imagined loudness time series model to theparticipant profile and the set of coefficients obtained byapplying the parent model to the recording intensity profile	Measure of how veridically participants imagined original intensity of each passage
Listening-intensitysimilarity	Distance between the set of coefficients obtained by applyingthe parent listening loudness time series model to theparticipant profile and the set of coefficients obtained byapplying the parent model to the recording intensity profile	Measure of how accurately participants rated original sounded intensity of each passage

#### Identification of correctly recalled profiles

Because the passages were long, participants were not always able to remember them in their entirety. The first stage of analysis, therefore, involved identifying participant profiles that corresponded to accurately recalled music so that this subset of participant data could be assessed for image veridicality. Dynamic time warping (DTW) was used to assess the accuracy of imagined tapping profiles, or their similarity to reference note onset profiles, as well as their length (see Appendix S1). Tapping profiles were composed of the series of IOIs between each tap. Where imagined tapping profiles were at least ^2^/_3_ the length of the recording profile and within two standard deviations of the mean measure of accuracy for a participant’s skill group, the participant was said to have remembered a sufficient quantity of the correct passage and their data were included in further analyses.

An original aim of the study had been to investigate imagery for expressive timing as well as loudness. However, the great difficulty many participants had in tapping out rhythms under both imagery and listening conditions meant that neither perceived nor imagined expressive timing could be meaningfully assessed. Tapping data are therefore only presented as the basis for a decision to retain or exclude participant loudness profiles.

#### Image-listening similarity

Rated loudness is subjective and a function of multiple acoustic parameters [4], [66], so variation in loudness profiles was expected between participants even in the listening condition. Using DTW, the similarity between imagined and listening loudness profiles was evaluated as a measure of how similar each participant’s subjective ratings were during these two conditions (see Appendix S1). This measure, hereafter referred to as ‘image-listening similarity’, indicates *how precisely* participants imagined loudness in as much of the passage as they were able to remember (as indicated by the length of the corresponding imagined tapping profile). A lower ‘image-listening similarity’ value corresponds to greater similarity between imagined and listening loudness profiles.

#### Recall

A second comparison of imagined and listening loudness profiles was made using DTW to assess *how much* of each passage participants were able to imagine. While image-listening similarity indicated similarity between imagined and listening loudness profiles in only as much of a passage as the participant tapped out correctly during the imagined tapping task, full-length imagined and listening loudness profiles were compared as an assessment of recall during the imagined loudness task (see Appendix S1). This measure is hereafter referred to as ‘recall’. A lower ‘recall’ value corresponds to better recall of the passage.

#### Image-intensity similarity

Time series modelling was used to evaluate how precisely each participant’s imagined loudness profiles reconstructed reference intensity profiles (see Appendix S2). The measure, representative of the distance between imagined loudness and recording intensity profiles, is referred to as ‘image-intensity similarity’ in subsequent analyses. A lower ‘image-intensity similarity’ value corresponds to greater similarity between imagined loudness and recording intensity profiles.

#### Listening-intensity similarity

The time series modelling process was repeated for listening loudness profiles (see Appendix S2), and the resulting distances make up the variable hereafter referred to as ‘listening-intensity similarity’. A lower ‘listening-intensity similarity’ value corresponds to greater similarity between listening loudness and recording intensity profiles.

#### Effect of musical expertise

As not all participants were able to meet the inclusion criteria for both stimuli, expertise groups based on OMSI score tertiles were calculated separately for each stimulus to ensure that sample sizes would be similar between groups (though not identical, since there were some ties in OMSI score). To determine whether expertise groups differed in how precisely their imagined loudness profiles reconstructed recording intensity profiles, three dependent variables and two potential covariates were entered into a MANCOVA for each of the experimental stimuli: (1) the measure of image-listening similarity, (2) the measure of recall, (3) the measure of image-intensity similarity, (4) the measure of listening-intensity similarity, as a potential covariate measure of listening ability and (5) the OSPAN score, as a potential covariate measure of working memory (Table 2).

## Results

### Excluded Trials

The proportion of trials excluded from the analysis was high due to the necessarily strict inclusion criteria (i.e. participants’ success at imagining loudness for a passage could not be assessed unless they could recall the passage in the first place). A total of 35 participant profiles were retained for the *Blue Danube* (20 excluded) and a total of 36 participant profiles were retained for *Habanera* (19 excluded). The high exclusion rates suggest that participants found recalling the long passages to be a difficult task despite the familiarisation period. Table 3 lists the number of profiles excluded for each of the two stimuli and the reason for each exclusion.

**Table 3:** Excluded trials.

Expertise group	Reason for exclusion			
	Insufficient length of tapping profile		Insufficient accuracy of tapping profile	
	BD	HA	BD	HA
Non-musician	14	14	0	1
Novice	3	3	1	0
Expert	1	0	1	1

*Note*. The number of trials excluded per group is listed for the excerpts from the *Blue Danube Waltz* (BD) and *Habanera* (HA). Across all participants, including those for whom data were excluded, the mean proportions of the excerpts recalled were 0.34 (14.9 s) for BD and 0.41 (14.2 s) for HA.

### Familiarity Ratings

Participants’ initial ratings of familiarity, completed at the start of the familiarisation phase, were compared with the ratings they provided at the time of the main experiment session to check whether familiarity improved as a result of the listening requirements. A t-test using data from both pieces showed that participants’ rated their familiarity with the passages significantly higher at the time of the main experiment than at the start of the familiarisation phase, *t*(69) = 4.71, *p*<.001. Familiarity ratings were also analysed to ensure that differences between expertise groups in imagery task performance were not attributable to differences in familiarity. Familiarity ratings from the start of the familiarisation phase and the time of the main experiment session, for all participants who met the inclusion criteria, were entered into a MANOVA with expertise group as the independent variable. This MANOVA showed no effect of expertise on familiarity, *F*(2, 41) = 0.46, *p = *.77. The number of times participants reported listening to each excerpt on CD was also equivalent across groups (non-musicians *M* = 13.3, *SD* = 4.4; novices *M* = 13.7, *SD* = 3.1; experts *M* = 13.0, *SD* = 2.8). These values were entered into an ANOVA with expertise group as the independent variable, and no effect of expertise on reported listening was found, *F*(2, 42) = 0.13, *p* = .88.

### Veridicality of Imagined Loudness

Figure S2 shows the intensity profiles and grand average imagined and listening loudness profiles for each piece. Grand average imagined loudness profiles were produced by calculating the mean slider position at each 250 ms time interval across participants’ shortened post-warping imagined loudness profiles; grand average listening loudness profiles were produced by calculating the mean slider position at each time interval across participants’ listening loudness profiles.

To see whether imagery task performance co-varied with either working memory span or listening abilities, correlations between the dependent variables and covariates were examined (Table 4). Recall and image-listening similarity were significantly correlated for both passages. Listening-intensity similarity, the measure of listening ability, did not differ systematically between expertise groups, and it did not correlate with any of the dependent variables, so it was not included as a covariate in the subsequent MANCOVAs. The OSPAN covariate correlated with recall for *Habanera* (*r*(34) = −0.37, *p = *.03), though not for the *Blue Danube*, and was retained as a covariate in the MANCOVAs. Since a higher OSPAN score corresponds to better working memory task performance and a lower score for recall corresponds to greater similarity between imagined and listening loudness profiles, a negative correlation between these variables suggests that people with larger working memory capacities tended to recall more of the *Habanera* excerpt.

**Table 4:** Correlations between measures of imagined loudness and covariates.

Stimulus		Image-intensity similarity	Image-listening similarity	Recall	Listening-intensity similarity
Blue Danube	Image-intensity similarity	–	–	–	–
	Image-listening similarity	0.07	–	–	–
	Recall	0.04	0.89**	–	–
	Listening-intensity similarity	0.02	0.03	0.07	–
	OSPAN	0.19	0.11	0.16	−0.02
Habanera	Image-intensity similarity	–	–	–	–
	Image-listening similarity	0.15	–	–	–
	Recall	−0.10	0.70**	–	–
	Listening-intensity similarity	−0.09	−0.19	−0.19	–
	OSPAN	−0.10	−0.22	−0.37**	0.11

*Note*. See Table 2 for definitions and descriptions of measure functions. Image-listening similarity and recall ability measures have been subjected to a logarithmic transformation.

* *p*<0.05.

** *p*<0.001.

#### Blue Danube

Logarithmic transformations were applied to the image-listening similarity and recall measures to approximate normality. A MANCOVA using the three dependent variables (image-intensity similarity, image-listening similarity, and recall) and OSPAN covariate was significant for Wilks’ lambda, *F*(3, 33) = 2.37, *p = *.02, with a significant main effect of expertise group, *F*(2, 33) = 3.21, *p = *.01. Planned comparisons indicated that at a Dunn-Sidak adjusted alpha of.03 [67], the difference between non-musicians and a combination of novices and experts, *F*(1, 30) = 5.96, *p = *.003, and the difference between experts and a combination of novices and non-musicians groups were significant, *F*(1, 30) = 5.82, *p = *.003. Image-listening similarity, recall and image-intensity similarity improved with increasing expertise (Table 5), though in terms of image-intensity similarity, the difference between non-musician and novice group means was negligible.

**Table 5:** Group means and standard deviations for measures of imagined loudness and covariates.

Stimulus	Expertise group	Mean distance (SD)				OSPAN^a^
		Image-intensity similarity	Image-listening similarity	Recall ability	Listening-intensity similarity	
Blue Danube	Non-musician *N* = 11 OMSI *M* = 39	7.06 (0.70)	7.32(2.8)	8.88(2.8)	6.82(0.78)	35.4(18.5)
	Novice*N* = 11OMSI *M* = 110	7.07(0.66)	4.26(4.9)	5.33(5.1)	7.15(0.72)	41(16.5)
	Expert*N* = 13OMSI *M* = 587	6.94(0.48)	2.62(2.2)	3.14(3.1)	6.56(0.71)	35.9(21.2)
Habanera	Non-musician*N* = 10OMSI *M* = 33	6.71(0.69)	3.90(3.9)	6.69(5.7)	7.16(0.48)	33.5(17.6)
	Novice*N* = 12OMSI *M* = 130	7.01(0.88)	2.51(4.2)	2.68(4.4)	6.95(0.61)	39.6(17.6)
	Expert*N* = 14OMSI *M* = 693	6.71(0.87)	2.02(2.4)	2.10(1.9)	7.07(0.55)	42.0(21.1)

*Note.* Mean OSPAN and OMSI scores differ between stimuli because different samples of participants met the inclusion criteria for each.

a Working memory covariate.

#### Habanera

Logarithmic transformations were similarly applied to the image-listening similarity and recall measures to approximate normality. A MANCOVA using the three dependent variables and the OSPAN covariate approached significance, *F*(3, 34) = 1.89, *p = *.067. The main effect of group was not significant, but planned comparisons indicated a difference between non-musicians and a combination of novices and experts that was marginally significant at an adjusted alpha of.03, *F*(1, 31) = 3.25, *p* = .036. Image-listening similarity and recall improved with increasing expertise (Table 5), though non-musicians and experts displayed similar image-intensity similarity.

## Discussion

In the literature on mental imagery, more attention has been paid to imagery for parameters that can be intrinsic to individual sound events, such as pitch, than parameters that are dynamic [32], [68], or meaningful because of how they change through time, such as melody or loudness [69]. The question of whether expressive loudness can be imagined and the relationship between expertise in music performance and the ability to imagine loudness were investigated with a set of tasks that required people to judge loudness under cued-imagery and listening conditions. Time series modelling and dynamic time warping were used to compare participants’ imagined loudness profiles to their listening loudness profiles and the original intensity profiles of the passages. Image- and listening-intensity similarity measures were comparable in magnitude, demonstrating a similarity between imagined and listening loudness. Participants in all expertise groups made imagined loudness judgements that were consistent with the loudness judgements they made while listening to the same passages of music, providing evidence that the ability to imagine loudness is widespread. Differences between groups in the accuracy with which loudness profiles were replicated during the imagery task suggest that the veridicality of imagined loudness may improve with increasing musical expertise.

As predicted, neither listening ability nor working memory capacity could account entirely for imagery task performance. Listening-intensity similarity did not correlate with any of the dependent variables for either stimulus or vary systematically with expertise across the two stimuli. It is unlikely that this was due to a ceiling effect related to participants’ high familiarity with the passages, given the difficulty that many participants had with recalling those passages. Since loudness is a perceptual correlate rather than a direct measure of intensity, some deviation between intensity and listening loudness judgments should be expected. The between-subject differences in listening loudness judgments that were observed emphasise the importance of comparing imagined loudness profiles not only to recording intensity profiles, but to listening loudness as well. The absence of a relationship between listening ability and imagery task performance suggests that greater perceptual acuity or attention to detail during music listening does not imply more effective retention. Though the lack of expertise effects on listening ability observed here is in contrast to some previous literature showing a positive correlation between musical expertise and perceptual acuity [21], [41], [43], in other studies, the predicted relationship between musical expertise and music perception or listening task performance has not been supported [40], [70], [71]. Further investigation is needed to clarify how perceptual acuity for individual parameters such as pitch, timbre, or duration relates to the perception of music in more naturalistic contexts, when multiple parameters are sounded in combination. Further investigation is also needed to investigate how music listening abilities relate to the effectiveness of encoding in memory, and how this process is affected by expertise, as the present study was not designed to address these questions.

The results of this experiment do not indicate a relationship between working memory capacity and musical expertise, since working memory capacity did not differ significantly between expertise groups. Furthermore, the results provide only limited evidence of a relationship between working memory capacity and musical imagery, since significant correlations between OSPAN score and imagery task performance were found for only one of the musical passages. For the *Blue Danube*, none of the dependent variables correlated significantly with OSPAN score, while for *Habanera*, there was a significant negative correlation between OSPAN score and the measure of recall. This suggests that participants with greater working memory spans tended to recall more of the *Habanera* excerpt. A relationship between working memory span and recall might have been masked by floor effects for the *Blue Danube.* The *Blue Danube* is a slow piece and in triple meter, while *Habanera* is faster and in duple meter. The main theme spans an equal number of bars in each piece, but this equates to a longer period of time for the *Blue Danube*, which may have made sustaining a mental image a more challenging task. Structural differences in tempo or meter or differences in familiarity may also have rendered *Habanera* easier to segment and retain in memory than the *Blue Danube*. On a broader scale, prior research suggests that instead of remembering long sequences of information, such as passages of music, in serial order, people remember them in meaningful chunks, with one chunk acting as a retrieval cue for the next [13], [39], [72], [73]. Recall fails when chunks are not reassembled properly in working memory [74]. The results of this experiment are consistent with the difficulty people are reported to have in recalling long sequences of music when the only retrieval cues available are imagined [39].

Of additional interest was the possibility that asymmetric differences in musical imagery ability would be observed between the three expertise groups. Though experts outperformed non-musicians on both pieces, novices did not differ reliably from the other two groups. Greater between-subject differences among novices in terms of musical abilities, combined with structural differences between the two pieces (e.g. length or meter), may have contributed to this higher degree of variability in novices’ imagery task performance. Continued study of the relationship between musical experience and understanding of musical structure is needed to determine how imagery ability is affected by their interaction.

### Musical Structure and Imagined Loudness

Musical structural groupings seemed to be reflected in some participants’ loudness profiles to a greater extent than in others’. Though most participants made loudness judgements at a global level, identifying only large-scale changes, examination of time series plots for imagined and listening loudness profiles suggests a minority made judgements at a local, phrasal level during one or both of the imagery and listening conditions (Figure S3). Whether people were to judge loudness changes at a local or global level was not specified in the experiment instructions, partly to avoid introducing a concept with which non-musicians might be unfamiliar. Though most of the participants whose profiles showed evidence of phrasing were experts, not all experts made loudness judgments at a phrasal level, and not all novices or non-musicians made loudness judgements at only a global level. This indicates that while the resolution at which loudness judgments tend to be made may vary as a function of expertise, it is not entirely dependent on prior musical experience. The factors underlying people’s ability and tendency to rate loudness at a local rather than global level while imagining and listening to music remain to be explored.

### Tapping Imagined Rhythms

A high proportion of participants failed to meet the inclusion criteria for analysis, which were based on the ability to recall at least two-thirds of a stimulus and accurately tap out its rhythm under cued-imagery conditions. High exclusion rates were expected, as it was necessary to exclude participants who could not recall enough of the music to be able to attempt the imagined loudness task with any possibility of accuracy. An inability to imagine the correct rhythm of recalled music may have impaired some participants’ performance on the imagined tapping task. However, as a number of studies have shown that people are capable of accurately imagining note durations [16], [17], [18], [19], it is more likely in this case that the passages were too long for most participants to recall without repeated prompting [39]. During debriefing, virtually all participants said they had trouble remembering all of the passages, suggesting that to achieve better recall, a more intensive familiarisation period or shorter musical passages should be used.

A related and surprising finding was the great degree of difficulty many of the participants with little or no musical experience had in tapping out rhythms while listening to the music. Beat-tapping tasks have been used in several studies to investigate musical imagery ability [23], [28], [75], and research has shown that non-musicians can accurately synchronise tapping with the beat of sounded music [70], [76], [77]; however, research on non-isochronous rhythm tapping ability in imagery and listening conditions and its relationship to musical expertise is lacking [78]. People have previously been found to predict beats more accurately when simultaneous auditory input is available than when it is not available [16]. Given such a finding, participants in the present study might have been expected to tap rhythms more accurately during the listening condition than during the imagery condition. Instead, for many participants, tapping task performance was poor under both imagery and listening conditions. It may be that some participants had difficulty identifying melodic lines in the multi-layered orchestral music that was used, though the experimental passages were selected in part because they had relatively simple rhythms and clear, well-known melodies to minimise this potential problem. Participants may also have had difficulty coordinating tapping movements and synchronising them with the sounds they were hearing or imagining. Listeners have previously been found to synchronise tapping more accurately with regular, mechanical versions of musical passages than versions performed with expressive timing [77]. In the current study, all of the musical excerpts were performed with expressive timing. Additional research is needed to determine how widespread the ability to tap non-isochronous musical rhythms is, and to determine how success on rhythm tapping tasks is influenced by simultaneous auditory input and the complexity of the music.

### Measuring Musical Imagery

Theories differ in how they conceptualise mental imagery [25], [26]. Depictive theories propose that images and their physical analogues are similar in form, such that relationships present in a physical stimulus are preserved when that stimulus is imagined [17], [27], [79]. Descriptive theories posit that mental representation occurs by way of a symbolic, language-like code [26]. It has been argued that evidence taken as support for depictive theories is often inconclusive, as successful performance on tasks assumed to require depictive imagery may be achieved by drawing on abstract knowledge about the world [25]. Some researchers have suggested studying brain activity in conjunction with behaviour to determine whether imagery task performance can be accounted for by participants’ abstract knowledge [7], [27], [80]. In a study by Wu et al. [7], for instance, people learned associations between visual cues (shapes) and sounded tones differing in loudness. Upon subsequent presentation of a visual cue, they were to imagine the corresponding tone, then compare the imagined tone to a sounded tone. EEG recordings revealed that the late positive complex previously found to relate to the generation of mental images was greater in amplitude when participants attempted to imagine loud tones than when they attempted to imagine quiet tones. This pattern mirrors that observed for the auditory-perception related N1 component, which is greater in amplitude when tones with high acoustic intensity are perceived than when tones with low acoustic intensity are perceived. The current experiment also offers support for the depictivist account of mental imagery. In studying mental imagery, it is a major challenge to discriminate between depictive and descriptive imagery and to provide evidence that one type was used to complete a particular task, while the other was not. In some previous research, imagery tasks have been developed that place heavy demands on memory, rendering successful task performance highly unlikely without the aid of depictive imagery [28]. With the method used in the present study, it is unlikely that participants could have mapped out loudness contours with high temporal precision had they not used a depictive image in which temporality was preserved. While it is possible that some descriptive knowledge was used in addition to this depictive image, achieving the same result by relying exclusively on descriptive knowledge of the pieces would have been extremely difficult, especially given that participants were not previously informed that the music would have to be recalled and were not instructed to attend to loudness or timing information until just before beginning the imagery task.

The method used in this experiment yielded results that were suggestive of a relationship between musical expertise and the ability to imagine loudness. Some non-musicians outperformed some experts, indicating that the tasks were accessible to people without musical training, despite the use of complex, naturalistic stimuli. As an early attempt to address the question of whether expressive loudness can be imagined, limits to the generalisability of the results from this experiment are acknowledged. While they suggest that expressive loudness can be imagined, they indicate that the ability to do so when imagining familiar music is also contingent on the ability to recall the music in the first place, then satisfy any motor demands the task may involve. The strict inclusion criteria filtered out participants who were unable to recall the passages or tap out their rhythms, potentially biasing the sample in favour of those with better memories for music. Perhaps the participants who failed to meet the inclusion criteria are less able to imagine loudness than the participants whose imagined loudness profiles were analysed. If this is the case, then the ability to imagine loudness may not be as widespread as the results of this study suggest.

Also, it might be argued that a study such as this assesses long-term memory for music rather than imagery ability. While imagined loudness data from the participants who were least capable of recalling the passages were not included in the analyses, it is possible that the differences in imagery task performance observed among the remaining participants were the result of differences in long-term memory rather than imagery ability. In our ongoing research, we are attempting to avoid this potential confound by asking participants to imagine short, novel music sequences containing changes in loudness instead of longer passages of familiar music [81].

### Conclusions

In most previous research on imagined loudness, the precision of imagery for the loudness of individual notes has been the focus of investigation, with conflicting results [7], [21], [24]. In the present study, non-musicians, novice musicians, and expert musicians made continuous loudness judgements that were consistent across imagined and listening conditions, consistent with the hypothesis that loudness can be imagined. Some support was offered for the predicted relationship between musical expertise and the ability to imagine the loudness of familiar music. Neither listening ability nor working memory capacity co-varied consistently with musical imagery ability or musical expertise.

Future research should investigate the possible mechanisms by which imagined relative loudness is achieved, which could include drawing on structural information stored in memory to reconstruct the auditory image, or a surface retrieval of specific loudness details. Further study may also indicate whether the ability to imagine music underlies the extraordinary precision and flexibility characteristic of expert music performance [82].

## Supporting Information

Figure S1 **Relationships among measures of imagined and listening loudness.**
(TIFF)

Figure S2 **Intensity and grand average imagined and listening loudness profiles.** Grand average profiles were calculated for the purpose of illustration only; comparisons between imagined loudness, listening loudness, and recording intensity profiles were only ever calculated within-subjects.
(TIFF)

Figure S3 **Recording intensity and sample novice and expert imagined loudness profiles.** The expert’s periodic slider adjustments (right) seem to correspond to intensity changes at a phrasal level while the novice (left) appears to have responded to the global increase in intensity without regard to phrasing.
(TIFF)

Figure S4 **Alignment achieved from the dynamic time warping of a non-musician’s imagined tapping profile with respect to the reference note onset profile for the passage from the** ***Blue Danube*** **.**
(TIFF)

Figure S5 **Alignment achieved from the dynamic time warping of a non-musician’s shorted imagined loudness profile with respect to the full-length listening loudness profile for the passage from** ***Habanera*** **.**
(TIFF)

Figure S6 **The alignment of a non-musician’s full-length imagined loudness profile (solid line) before and after dynamic time warping with respect to the listening loudness profile (dotted line) for the passage from** ***Habanera*** **.**
(TIF)

Figure S7 **Parent model forecasts and sample participants’ differenced imagined loudness profiles for the** ***Blue Danube*** **and** ***Habanera*** **.** Time is measured in 100ms intervals. These plots provide an indication of how accurately individual participants’ imagined loudness profiles are forecast by the parent models. A period of approximately 30 events precedes the start of the forecasts because the models included intensity lags of 30.
(TIFF)

Appendix S1 **Analyses conducted using dynamic time warping.**
(DOCX)

Appendix S2 **Analyses conducted using time series modelling.**
(DOCX)
