# Supplementary figures and images for: Musical Expertise and the Ability to Imagine Loudness
Source: PLoS One. 2013 Feb 27;8(2):e56052. doi: 10.1371/journal.pone.0056052 (PMC3584072; doi:10.1371/journal.pone.0056052)

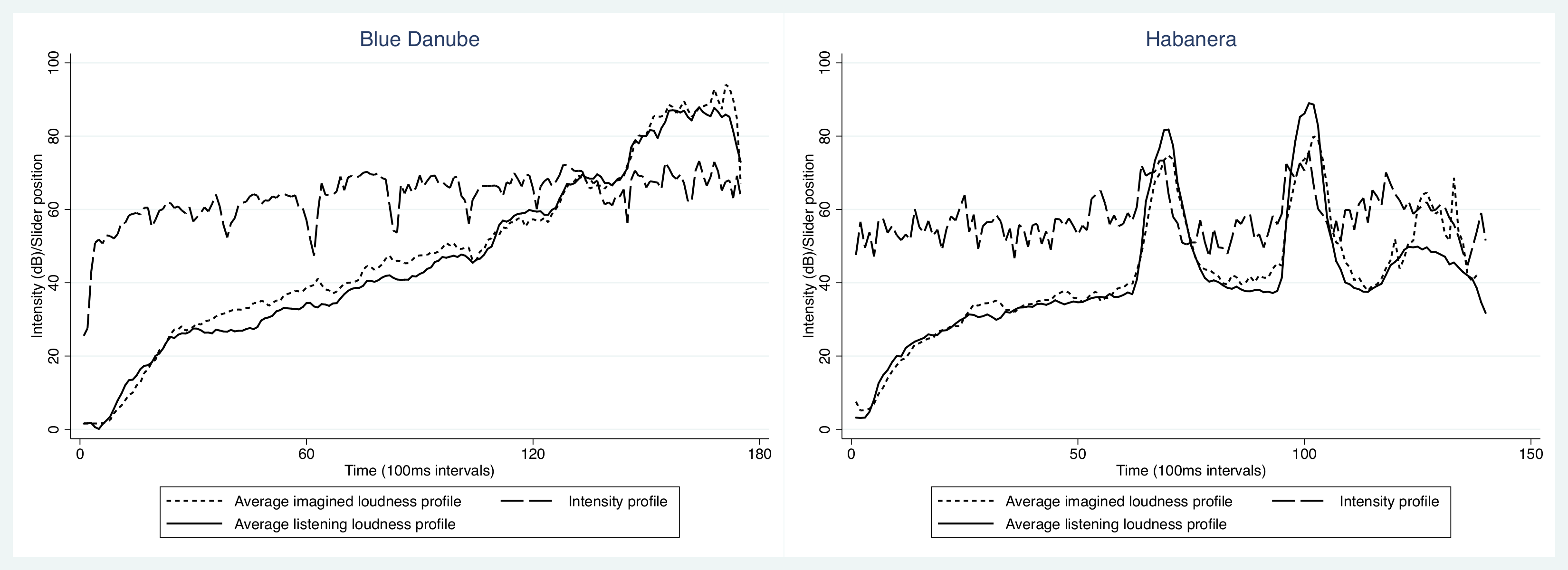

Supplement: Figure S2 — Intensity and grand average imagined and listening loudness profiles. Grand average profiles were calculated for the purpose of illustration only; comparisons between imagined loudness, listening loudness, and recording intensity profiles were only ever calculated within-subjects. (TIFF) [file pone.0056052.s002.tif]

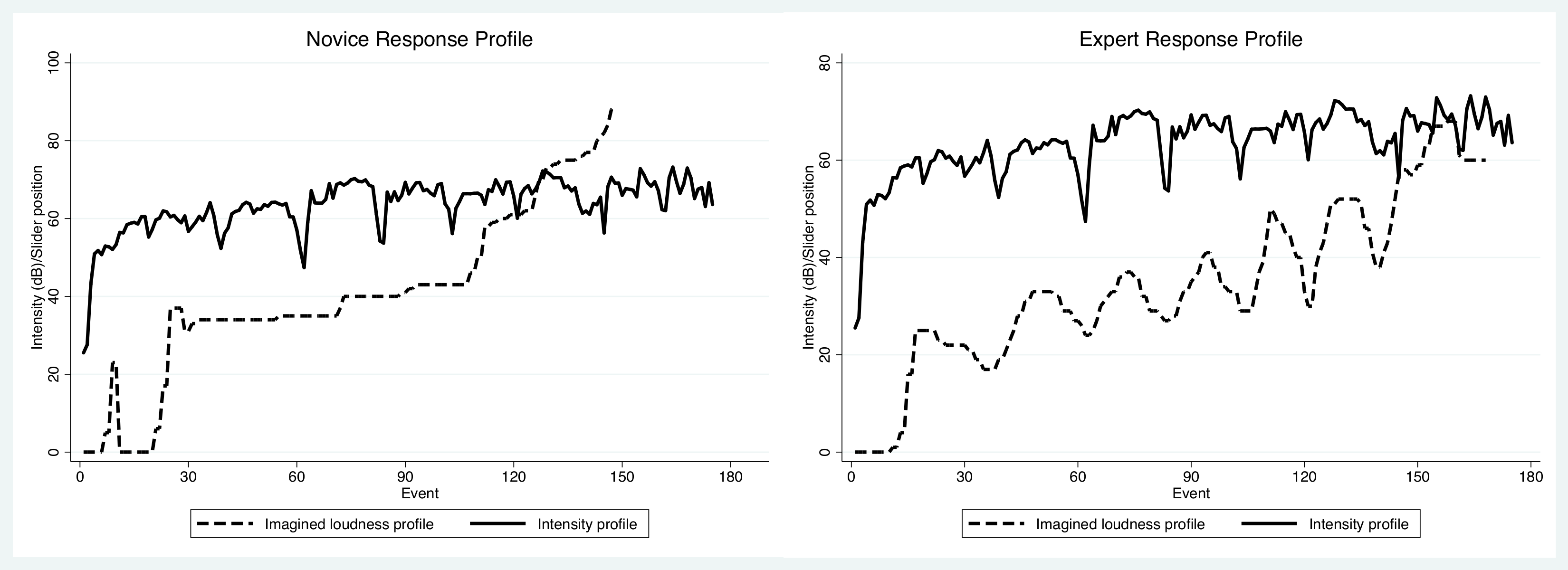

Supplement: Figure S3 — Recording intensity and sample novice and expert imagined loudness profiles. The expert’s periodic slider adjustments (right) seem to correspond to intensity changes at a phrasal level while the novice (left) appears to have responded to the global increase in intensity without regard to phrasing. (TIFF) [file pone.0056052.s003.tif]

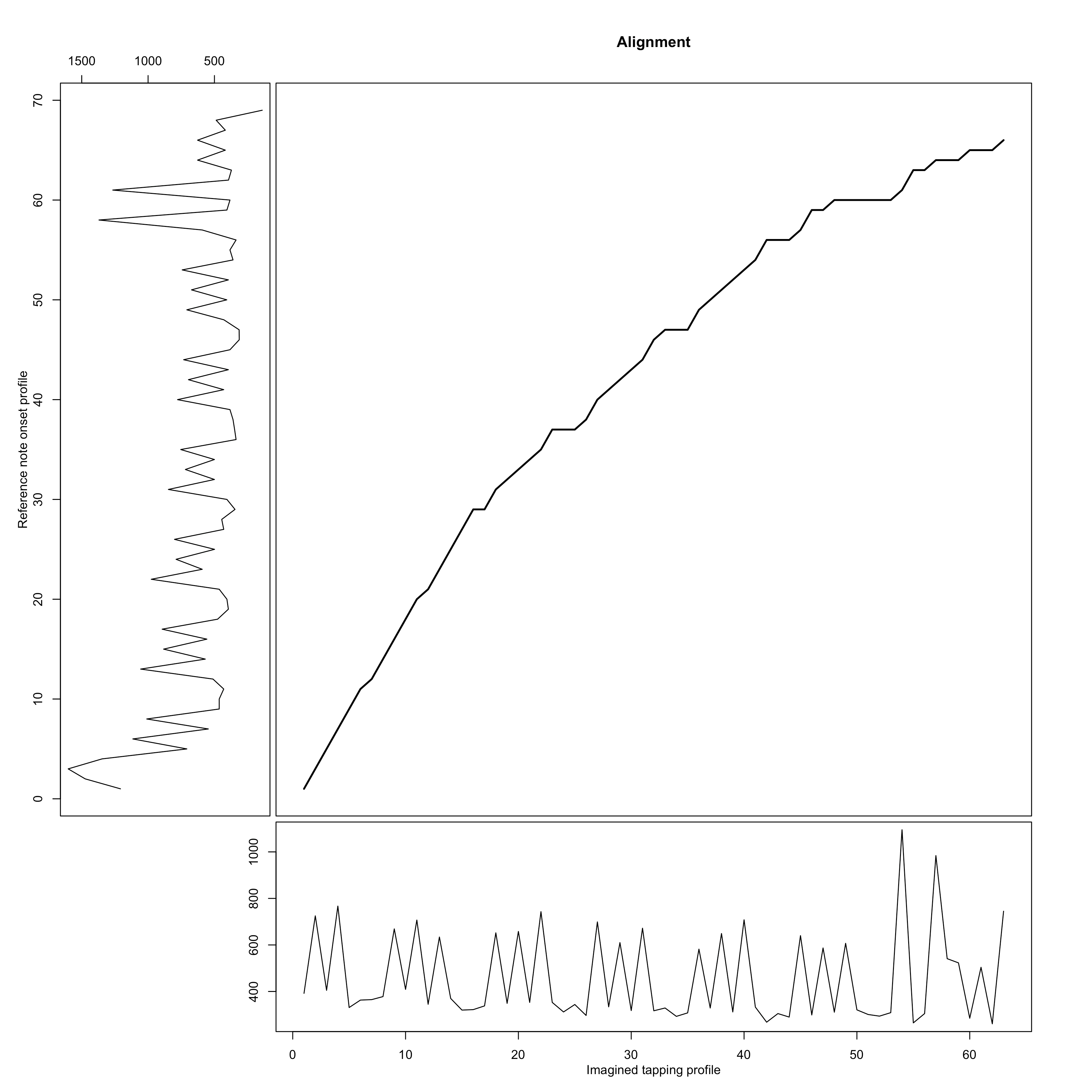

Supplement: Figure S4 — Alignment achieved from the dynamic time warping of a non-musician’s imagined tapping profile with respect to the reference note onset profile for the passage from the Blue Danube . (TIFF) [file pone.0056052.s004.tif]

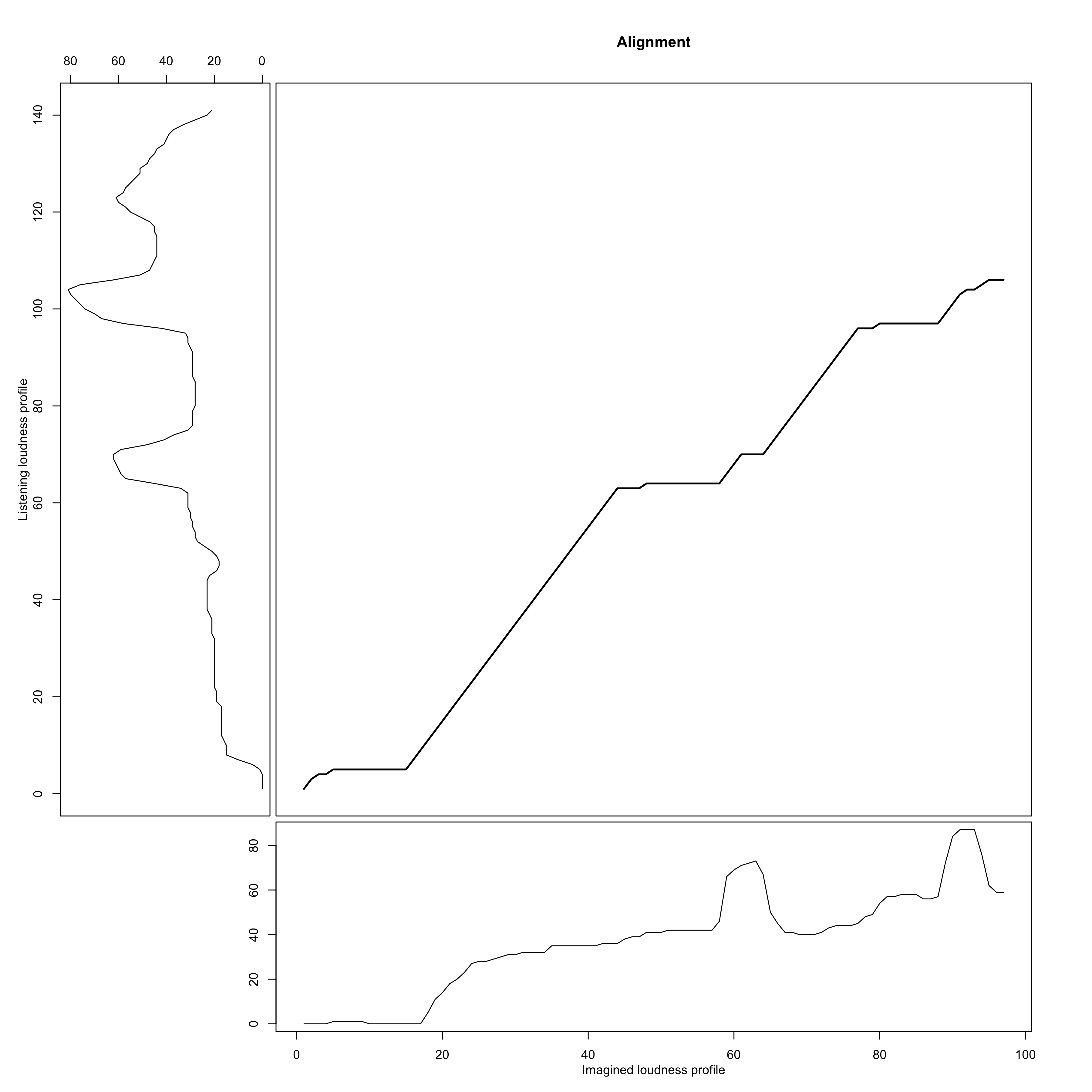

Supplement: Figure S5 — Alignment achieved from the dynamic time warping of a non-musician’s shorted imagined loudness profile with respect to the full-length listening loudness profile for the passage from Habanera . (TIFF) [file pone.0056052.s005.tif]

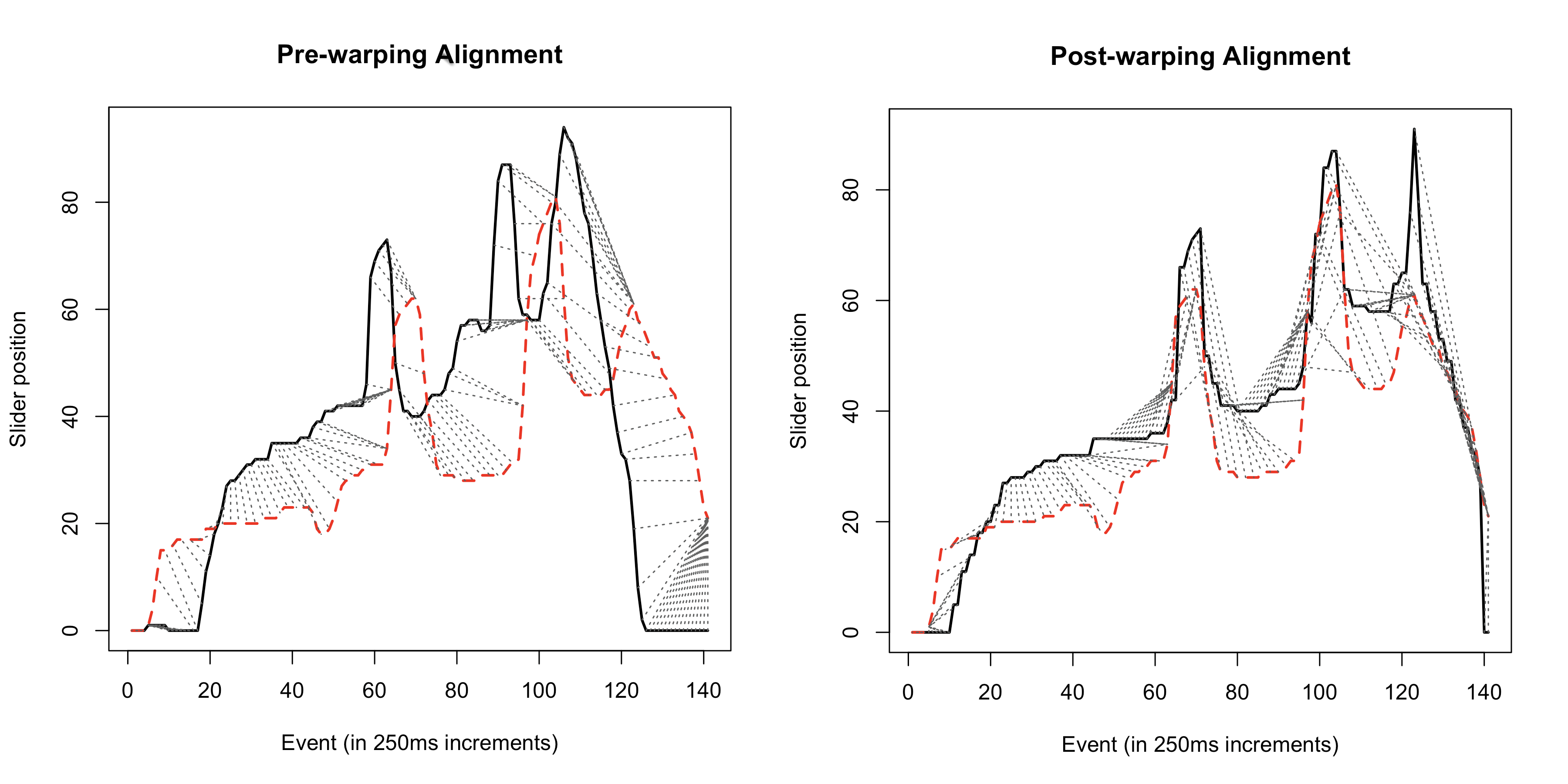

Supplement: Figure S6 — The alignment of a non-musician’s full-length imagined loudness profile (solid line) before and after dynamic time warping with respect to the listening loudness profile (dotted line) for the passage from Habanera . (TIF) [file pone.0056052.s006.tif]

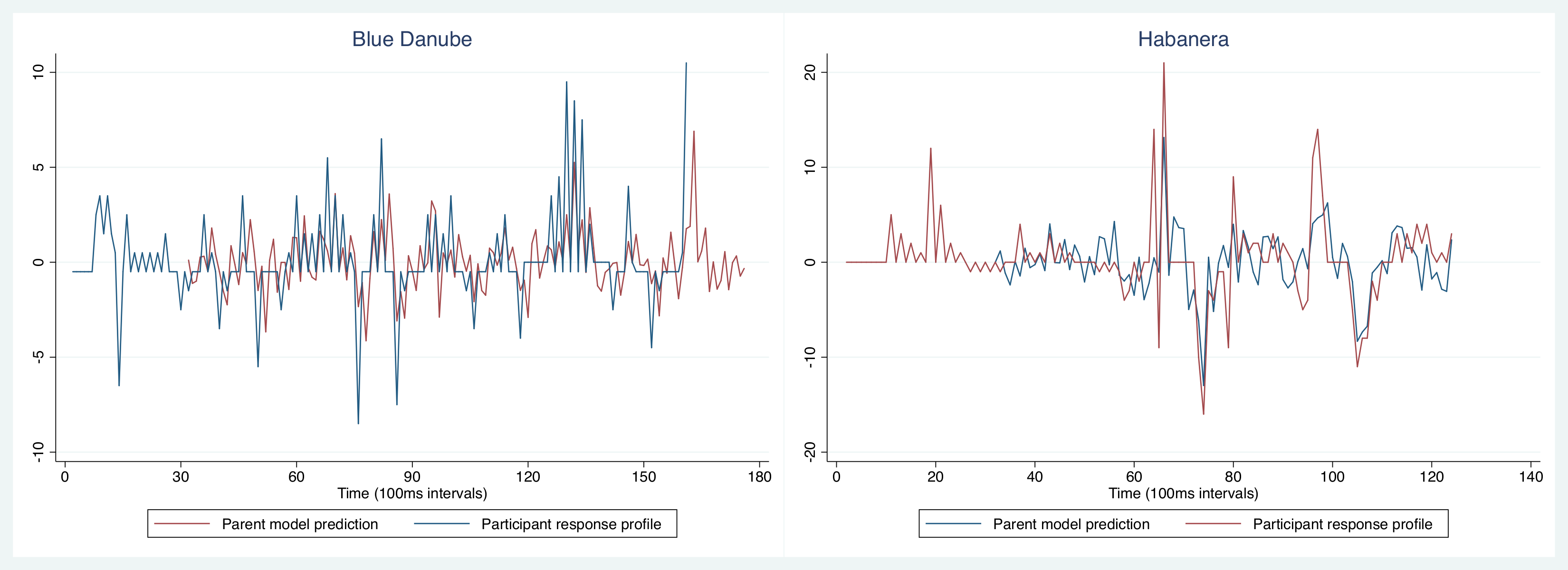

Supplement: Figure S7 — Parent model forecasts and sample participants’ differenced imagined loudness profiles for the Blue Danube and Habanera . Time is measured in 100ms intervals. These plots provide an indication of how accurately individual participants’ imagined loudness profiles are forecast by the parent models. A period of approximately 30 events precedes the start of the forecasts because the models included intensity lags of 30. (TIFF) [file pone.0056052.s007.tif]
