# Supplementary material for: Musical Expertise and the Ability to Imagine Loudness
Source: PLoS One. 2013 Feb 27;8(2):e56052. doi: 10.1371/journal.pone.0056052 (PMC3584072; doi:10.1371/journal.pone.0056052)
Supplement: Appendix S1 — Analyses conducted using dynamic time warping. (DOCX) [file pone.0056052.s008.docx]

Appendix S1

**Analyses Conducted Using Dynamic Time Warping**

Dynamic time warping was conducted in R [1] to determine how precisely each participant’s imagined timing profiles reconstructed the original recording timing profiles and to assess the similarity between imagined and listening loudness profiles. This statistical procedure stretches and compresses a test data series along one dimension so that it aligns as closely as possible with a reference series (Figure S1-1). Both global and partial alignments can be calculated. In a global alignment, a full test series is mapped onto a full reference series, with the first and final test series points always matched to the first and final reference series points. In a partial alignment, only the subsection of the test series that best minimises the post-warping normalised distance (see below for explanation) between test and reference series is mapped onto the reference series. This subsection was assumed to correspond to as much of a passage as a participant was able to remember, and thereby varied in length from only a few events to the entire passage. To compute the partial alignments in the present analyses, end-point constraints were relaxed, allowing excess data to be removed from the beginning or end of test series and the post-warping distance between test and reference series to be minimised. The analysis, therefore, did not distinguish between a participant who was only able to recall the beginning of a passage and a participant who forgot an early section, but then went on to recall later sections correctly.

Partial alignments were used to measure 1) the recall of experimental passages and 2) image veridicality. Recall of experimental passages was represented by the number of events in the optimal post-warping curve and veridicality of images was represented by the post-warping distance between test and reference series. In other words, large-scale deviations in tapped rhythm or slider movements, corresponding to points at which participants failed to remember any more of the passage, affected the measure of recall, while localised deviations affected the measure of image veridicality. A participant who was only able to remember the first three-quarters of the *Blue Danube* but imagined it with a high degree of accuracy, for example, would have fewer events in their optimal curve and a smaller post-warping normalised distance than a participant who was able to remember the entire passage but imagined it less accurately.

An asymmetric step-pattern was used for all alignments. Step-patterns dictate the permissible transitions between matched test and reference profile pairs, or the amount of stretch and compression allowed at any given point. The asymmetric step-pattern allows each point in the test series to be matched to only one point in the reference series, and was chosen for use in these analyses because it did not excessively shorten test series in determining optimal warping curve length, as some other commonly used step-patterns did. The alignments achieved under these constraints are thus only one of several possible alignments, and not necessarily the ideal solution for every comparison despite being the most successful in general.

The normalised distances used in the present analyses represent the average distance between test and reference series per step, enabling test series length to be taken into account and avoiding the advantage otherwise given to shorter post-warping curves.

**Identification of correctly recalled profiles.** Imagined tapping profiles were warped with respect to reference note onset profiles using this partial alignment, and the resulting measures were used as the basis for retaining or excluding data series. If the optimal fit was shorter than ^2^/_3_ of the recording profile, or if the post-warping normalised distance between imagined and recording profiles was greater than two standard deviations above the mean for the participant’s skill group, the participant was said to have failed to remember a sufficient quantity of the correct passage and the data series was rejected from further analysis.

**Image-listening similarity and recall.** Two separate analyses were conducted on each imagined loudness profile to assess 1) similarity in loudness judgments between imagined and listening conditions and 2) recall. First, it was assumed that participants were able to imagine approximately the same portion of each passage in both tapping and loudness imagery conditions. Excess data were therefore removed from the end of imagined loudness profiles so that shortened imagined loudness profiles and post-warping imagined tapping profiles spanned the same total duration. If, during the imagined tapping condition, for example, a person successfully tapped out the rhythm for the first 20 seconds of *Habanera* but failed to remember the last 15 seconds, then only the first 20 seconds of slider positions recorded during the imagined loudness condition were retained. Shortened imagined loudness profiles were then time-warped with respect to full-length listening loudness profiles, with end-point constraints relaxed such that the optimal alignment was calculated without necessarily matching the final point of the imagined loudness profile with the final point of the listening loudness profile (Figure S1-2). The difference between pre- and post-warping normalised distances separating shortened imagined and full-length listening loudness profiles comprises the measure referred to as ‘image-listening similarity’.

Full-length imagined loudness profiles were also warped with respect to full-length listening loudness profiles, again with end-point constraints relaxed (Figure S1-3). The difference between pre- and post-warping normalised distances separating full-length imagined and listening loudness profiles, referred to as ‘recall’ in subsequent analyses, indicated how much of each passage participants were able to imagine.

**References**

1. Giorgino T (2009) Computing and visualizing dynamic time warping alignments in R: The dtw package. J Stat Softw 31: 1-24.
