# Supplementary material for: Musical Expertise and the Ability to Imagine Loudness
Source: PLoS One. 2013 Feb 27;8(2):e56052. doi: 10.1371/journal.pone.0056052 (PMC3584072; doi:10.1371/journal.pone.0056052)
Supplement: Appendix S2 — Analyses conducted using time series modelling. (DOCX) [file pone.0056052.s009.docx]

Appendix S2

**Analyses Conducted Using Time Series Modelling**

**Image-intensity and listening-intensity similarity.** Time series modelling was used to compare each participant’s imagined and listening loudness profiles with the intensity profiles of the recordings [1]. Time series analysis was first conducted on all post-warped imagined loudness profiles containing at least 65 events of sampled data. Each participant response and recording intensity profile was differenced once in order to achieve stationarity. A time series can be considered stationary if its mean and variance are constant across time points (i.e. there are no substantial trends). Differencing involves calculating the difference between successive values in a time series, and produces a new series with one fewer event and a lesser degree of autocorrelation. For each stimulus piece, a parent ARIMAX model (i.e. a model including endogenous, exogenous and autoregressive components) was produced based on endogenous and exogenous components most often included in models for the imagined loudness series of individual participants. Endogenous components represented the contribution of previous slider positions to the current slider position, while the exogenous component represented the contribution of the reference recording intensity. Table S2-1 lists the components used in the imagined and listening parent models for each piece. The 65 event minimum criterion for post-warped imagined loudness profiles was imposed at this stage because at least 60-80 events proved necessary to compare all participant profiles to a parent model that did not over-specify the shortest profiles or under-specify the longest profiles: profiles shorter than 65 events could not be compared to the parent models without modifying the models to exclude intensity lags above 20. The fit of each participant’s imagined loudness profile to the parent model was evaluated, producing a vector of model coefficients for each participant. Figure S2-1 illustrates the parent model forecast and sample participants’ differenced imagined loudness profiles for the two pieces. Finally, the Mahalanobis distance between each vector and that of the parent model applied to the recording intensity profile was measured. Mahalanobis distance, unlike Euclidian distance, takes into account correlations and the density patterns of non-spherically distributed data. It is scale-invariant and often used for calculating either the distance between the mean of a distribution and individual data points or the distance between the mean of an unknown distribution and the mean of a known one [2,3]. These distances comprise the measure referred to as ‘image-intensity similarity’.

This process was then repeated for listening loudness profiles. A parent ARIMAX model was produced based on individual participant listening loudness models. The fit of each participant’s listening loudness profile to the parent model was evaluated and the Mahalanobis distance between each vector and that of the parent model applied to the recording intensity profile was measured. These distances comprise the measure referred to as ‘listening-intensity similarity’.

**References**

1. Dean RT, Bailes F (2010) Time series analysis as a method to examine acoustical influences on real-time perception of music. Empir Musicol Rev 5: 152-175.

2. Farber O, Kadmon R (2003) Assessment of alternative approaches for bioclimatic modeling with special emphasis on the Mahalanobis distance. Ecol Modell 160: 115-130.

3. Mahalanobis P (1936) On the generalized distance in statistics. Journal of the Asiatic Society of Bengal 26: 49-55.
